# Mathematical modeling of tumor-associated macrophage interactions with the cancer microenvironment

**DOI:** 10.1186/s40425-017-0313-7

**Published:** 2018-01-30

**Authors:** Grace Mahlbacher, Louis T. Curtis, John Lowengrub, Hermann B. Frieboes

**Affiliations:** 1 Department of Bioengineering, University of Louisville, Lutz Hall 419, Louisville, KY 40208 USA; 2 Department of Mathematics, University of California, 540H Rowland Hall, Irvine, CA 92697 USA; 3 Department of Biomedical Engineering, University of California, Irvine, CA USA; 4 Department of Bioengineering, University of Louisville, Lutz Hall 419, Louisville, KY 40208 USA; 5 James Graham Brown Cancer Center, University of Louisville, Louisville, KY USA; 6 Department of Pharmacology & Toxicology, University of Louisville, Louisville, KY USA

**Keywords:** Cancer immunotherapy, Cancer metastasis, Tumor-associated macrophages, Tie2 expressing macrophages, Mathematical modeling, Computational simulation

## Abstract

**Background:**

Immuno-oncotherapy has emerged as a promising means to target cancer. In particular, therapeutic manipulation of tumor-associated macrophages holds promise due to their various and sometimes opposing roles in tumor progression. It is established that M1-type macrophages suppress tumor progression while M2-types support it. Recently, Tie2-expressing macrophages (TEM) have been identified as a distinct sub-population influencing tumor angiogenesis and vascular remodeling as well as monocyte differentiation.

**Methods:**

This study develops a modeling framework to evaluate macrophage interactions with the tumor microenvironment, enabling assessment of how these interactions may affect tumor progression. M1, M2, and Tie2 expressing variants are integrated into a model of tumor growth representing a metastatic lesion in a highly vascularized organ, such as the liver. Behaviors simulated include M1 release of nitric oxide (NO), M2 release of growth-promoting factors, and TEM facilitation of angiogenesis via Angiopoietin-2 and promotion of monocyte differentiation into M2 via IL-10.

**Results:**

The results show that M2 presence leads to larger tumor growth regardless of TEM effects, implying that immunotherapeutic strategies that lead to TEM ablation may fail to restrain growth when the M2 represents a sizeable population. As TEM pro-tumor effects are less pronounced and on a longer time scale than M1-driven tumor inhibition, a more nuanced approach to influence monocyte differentiation taking into account the tumor state (e.g., under chemotherapy) may be desirable.

**Conclusions:**

The results highlight the dynamic interaction of macrophages within a growing tumor, and, further, establish the initial feasibility of a mathematical framework that could longer term help to optimize cancer immunotherapy.

## Background

The role of tumor-associated macrophages (TAM) in tumor growth [1] and treatment response [2, 3] has been the subject of increased study. What has become clear is that populations of TAM are diverse in both phenotype and lineage [4, 5]. An increased presence of macrophages at a tumor lesion site is generally correlated with poor prognosis. The phenotypic range of TAM contributes in various ways to the tumor progression [1], including tumor-promoting and tumoricidal phenotypes which reflect the conflicting cues within the tumor environment.

The M1 extreme of the macrophage activation spectrum is commonly associated with inflammatory responses and tumoricidal activity driven by the expression of inducible nitric oxide (NO) synthase and the release of proinflammatory cytokines, which encourage tumor cell apoptosis [6, 7]. Its presence in the tumor microenvironment is correlated with reduced angiogenesis required to supply the increased tumor metabolic needs, and thus reduced tumor growth and survival [5, 8]. The relative proportion of the M1 macrophages generally decreases with tumor progression. The M1 subtype is identified by surface receptors CD14^++^CD16^−^ [6].

The M2 or alternatively activated macrophages encompass a broader family of macrophages involved in tissue healing under normal conditions. Within the tumor microenvironment, they are recruited for tumor progression [1], and generally comprise a larger portion of the TAM in advanced tumors [1, 9]. Hypoxia-induced factors such as VEGF-A, endothelin-2, and interleukin-10 secreted in the tumor environment encourage differentiation towards the M2 phenotype [10]. Within the tumor microenvironment, M2 s secrete factors such as TGF-β1 which facilitates cancer cell proliferation [5, 11], VEGF-A which promotes angiogenesis and recruits additional macrophages, and MMP-9 which facilitates angiogenesis by degrading the extracellular matrix [1]. The proportion of M2 macrophages in the microenvironment tends to increase with tumor progression. The M2 subtype is identified by surface receptors CD14^dim^CD16^+^ [5].

While the immune response may begin as primarily tumoricidal, with macrophages of the M1 or classically activated type targeting tumor cells, cytokines secreted by the tumor exploit the relatively fluid phenotype of the TAM to promote tumor growth and survival [8] via the M2 subtype. A third, more recently discovered subtype called Tie2 expressing macrophage (TEM) develops from a distinct precursor, and displays unique and non-redundant behaviors highly relevant to tumor-induced angiogenesis [12, 13] and post-ischemic recovery [14]. TEMs can be identified by the expression of Tie2 on their surface which is also found on the endothelial cells of blood vessels, where they are integral to angiogenic pathways and development [6, 15]. Tie2 is a transmembrane tyrosine-protein kinase receptor regulating angiogenesis, including endothelial cell survival, migration, and proliferation.

Recent research indicates that TEMs are recruited to the tumor microenvironment at an early phase of development. There, they are believed to play a pivotal role in tumor neovascular development by activating the “angiogenic switch” – a transition that occurs when a tumor begins to recruit nearby vasculature to supply its increased metabolic demands [16]. The critical role of TEMs in tumor angiogenesis and vascular remodeling [12, 13, 17] was shown by increased TEM infiltration following administration of anti-angiogenic agents [18] as well as the blocking of the angiogenic factor angiopoietin-2 (Ang2), a Tie2 ligand associated with activated endothelial cells, leading to tumor vasculature regression and arrested tumor progression [19].

The chief contribution of TEMs to tumor progression appears to be facilitation of angiogenesis through structural and paracrine support. The macrophage’s titular receptor, Tie2, binds the growth factors angiopoietin 1 and 2. In addition to having a direct chemotactic effect on the TEMs [20] interactions with angiopoietins lead to the upregulation of several factors necessary to angiogenic processes, including MMP-9, CTSB, and IL-10, not dissimilar to the role of the M2 subtype [20, 21]. However, TEMs have a more multifaceted involvement in angiogenesis [12]. As well as upregulating these factors in TEMs, Ang2 acts as a chemoattractant, causing the TEMs to congregate along the abluminal side of vessels [22]. Here, TEMs are thought to directly facilitate vessel sprouting by providing both a structural scaffold and paracrine support for endothelial sprouts, aiding in their growth [23], and preventing collapse due to the high hydrostatic pressure associated with the tumor microenvironment [15]. As a tumor grows and its metabolic needs increase, TEMs continue to fill a supportive role in the growth and maturation of the neovasculature that supplies it with nutrients and oxygen [15]. In a 2008 study by De Palma et al. comparing a tumor model with an intact and TEM-knockout population of tumor associated macrophages, the tumors with an intact population showed a four-fold increase in vascular development in comparison to the TEM-ablated population [12].

In addition to their more direct roles in facilitating neovascular development, TEMs also contribute to the cocktail of other tumor-friendly cytokines in the microenvironment. IL-10 is an immune cytokine secreted from most leukocytes, including macrophages, as well as from tumor cells themselves [24]. It has pleiotropic effects in the tumor microenvironment, being implicated in both suppression of tumorigenic cytokines such as IL-6 [25], and in enhanced immune escape, poor prognosis, and advanced cancer stage [25]. While IL-10 is known to be upregulated in several cancer types, including breast cancer [25], a consensus has yet to be reached on whether it is a definitive indicator of tumor progression and patient prognosis, as some studies have suggested that its overexpression leads to subsequent immune rejection of the tumor [26]. IL-10 also plays a role in inducing infiltrating monocytes to adopt the tumorigenic M2 phenotype [5]. Tie2-expressing macrophages are known to secrete IL-10, and thus may contribute to the increased ratio of M2 to M1 macrophages in the tumor microenvironment.

The interplay of the various macrophage subtypes within the changing tumor microenvironment presents a relevant and challenging task which may benefit from a systems analysis perspective. To this end, recent mathematical modeling and computational simulation work [11] has evaluated the uptake of nanoparticles by macrophages in the tumor microenvironment to gain insight into implications for cancer treatment and drug delivery. In this study, we develop a mathematical framework to systematically evaluate the role of TEMs in relation to M1 and M2 macrophage phenotypes on the growth of vascularized tumor lesions. The TEMs are simulated to differentiate from vascular-extravasated monocyte precursors, congregate around the abluminal side of the vasculature in response to a chemoattractant gradient, secrete cytokines which favor differentiation of a separate angiogenic macrophage subtype [27], and significantly upregulate angiogenic activity and survival of neovasculature.

## Methods

Previous mathematical modeling work has explored critical aspects of TAM activity. Owen and Sherratt [28, 29] presented a model in which macrophages entered the tumor environment to selectively target tumor cells. Later models were developed to simulate macrophages primed to destroy cancer cells on contact [30] or by drug delivery [31]. In [32] it was shown that effective macrophage targeting of hypoxic tumor cells would benefit from non-cell-cycle dependent drugs or limited-diffusivity. In [33] it was found that the combination of conventional and macrophage-based therapies using magnetic nanoparticles could be synergistic. In [34] the role of tumor macrophage hypoxia inducible factors (HIFs) in chemotherapy effectiveness was evaluated. Recently, a model exploring the efficacy of nanoparticle albumin-bound-paclitaxel (nab-PTX) using macrophages as a delivery vehicle to target hypovascularized cancer lesions was developed [11].

The computational model in this study builds upon this recent work simulating generic TAM activity in the tumor microenvironment [11], in which a breast cancer lesion metastasized to the liver was simulated – under conditions that are known to favor the recruitment of TAM [35]. In [11], macrophages were utilized as a drug vector, and their performance was evaluated experimentally and via computational simulation. In [36], this system was expanded to include M1 and M2 macrophage variants in order to gauge their shifting role in the tumor response to the drug therapy. Here, we do not assume that drug is vectored by macrophages, and focus solely on the effects of the various macrophage population subtypes on the tumor lesion progression.

Briefly, the model is composed of a tumor lesion in a 2D grid of preexisting vasculature as previously described in [11, 36–39]. A spatial component is included in order to model the movement of macrophages as well as the transport of cytokines, and other substances in the tumor microenvironment. We define macrophage subtypes derived from bone marrow precursors, namely, the M1 and M2 subtypes, and add the TEM subtype as a third population that promotes angiogenesis for tumors lesions. Given that the monocytes are not biologically active in the model, the simplifying assumption is made that Tie2 expressing macrophages differentiate in the tumor microenvironment from the same type of monocyte precursor as the M1 and M2 macrophages. The effects of the TEM phenotype on the environment are modeled with the following characteristics:

- Increasing differentiation of Tie2-expressing macrophages from a monocyte precursor with tumor progression
- Semi-stochastic movement of TEMs along a chemoattractant gradient (Ang2) secreted by the peritumoral vasculature, as well as a macrophage attractant from the hypoxic regions of the tumor.
- The representative protein released by the TEMs is modeled after IL-10 to examine the effects of cytokine release in the context of immunomodulatory activity.
- Increased M2 differentiation in response to TEM-eluted IL-10 in the system
- Increased angiogenesis and resilience of tumoral neovasculature due to local TEM presence

### Tumor growth

The tumor growth model is based on Macklin et al. [37] and builds upon recent work [11, 36, 38, 39]. Simulation of tumor growth begins with a small lesion in a 2D grid of blood vessels representing a regularly-spaced (250 μm) capillary grid. The tumor progression is modeled in discrete time increments, enabling evaluation, updating, and recording of the tumor conditions. Advection of the tumor and advancement of its boundary are subject to changes in the microenvironment such as fluid pressure, diffusion of hypoxic proteins and other angiogenic factors, and concentration of oxygen, glucose and other vital nutrients (here, simplified as oxygen only). Altogether, the tumor microenvironment may be described in four regions based on oxygen and proliferation levels. These are:

- Necrotic region, Ω_N_, in which oxygen levels are insufficient for viability.
- Hypoxic region, Ω_H,_ in which oxygen levels are sufficient for viability but not proliferation.
- Proliferating region, Ω_P_, in which oxygen levels are sufficient for proliferation.
- Normal (non-tumoral) tissue.

Tumor boundary advancement with velocity *v*_*c*_ through the porous extracellular matrix of the surrounding normal tissue is based on Darcy’s law [37]:

1 $$ {v}_c=-\mu \nabla P+{\chi}_E\nabla E $$

where *μ* is tissue mobility, encompassing the roles of cell-cell and cell-matrix adhesion, *P* is the oncotic pressure, χ_*E*_ is haptotaxis, and *E* is the density of the extracellular matrix. Via a simplifying assumption of uniform density *E* in the proliferating tumor region, the relationship between velocity change and tumor growth is [37]:

2 $$ \nabla \cdot {v}_c={\lambda}_p $$

where *λ*_*p*_ is the non-dimensionalized net tumor proliferation rate (described below).

As oxygen falls below a threshold for proliferation regions distal from vasculature, hypoxic tissue regions develop and release tumor angiogenic factors (TAF). The TAF diffuse outward through the tumor and into the surroundings, where they trigger endothelial cell sprouts in the peritumoral vascular grid as well as vascular extravasation of macrophages, analogous to the action of VEGF on macrophage recruitment to the tumor [40]. If oxygen falls below a vital threshold, necrotic tissue develops within the tumor. The tumor model main parameters are listed in Table 1.

**Table 1:** Tumor model main parameters and associated values

Parameter	Value	Reference
Tumor tissue threshold for hypoxia	0.5750	Calibrated to match [11]
Tumor tissue threshold for necrosis	0.5325	Calibrated to match [11]
Oxygen diffusivity	1 (^*^)	[39]
Oxygen transfer rate from vasculature	5 (^*^)	[39]
Oxygen uptake rate by proliferating tumor cells	1.5 (^*^)	[39]
Oxygen uptake rate by hypoxic tumor cells	1.3 (^*^)	[39]
Oxygen uptake rate by tumor microenvironment	0.12 (^*^)	[39]
Oxygen decay rate	0.35 (^*^)	[39]

(^*^) Value is non-dimensionalized by the diffusivity of oxygen [68] (1 × 10^−5^ cm^2^ s^−1^)

### Angiogenesis and vascular development

The angiogenesis model, adapted from [37, 39, 41], describes the development and maturation of a tumor-induced neovascular network, blood flow through the network and the mechanical and chemical effects of tumor proliferation on the growth, maturation, flow, flux, and collapse of the surrounding vasculature. The vasculature is simplified to a grid, from which irregular new vessels sprout and grow in response to gradients of factors and pressures produced by the tumor tissue. We simulate a highly vascularized organ microenvironment, e.g., as is the case for the lung or the liver, providing an environment conducive to metastases formation.

As the tumor grows within the vascularized microenvironment, cancer cells may experience heterogeneous access to oxygen, glucose, and growth factors, which may depend on distance from the nearest vascular source as well as interstitial and oncotic pressures. Each vessel sprout grows semi-stochastically, with the probability of growing in one of four directions weighted by the presence of the TAF gradient produced by the hypoxic tissue. The sensitivity of the vascular growth is increased in response to contact with factors secreted by the TEMs. The magnitude of this response was calibrated to correlate with the four-fold increase in vasculature surface area found to result from TEM-eluted factors by De Palma et al. [12].

The change *∆R* in radii *R* of the vessels is modeled according to pressures imposed by the movement of fluid within them [37, 41–43],

3 $$ \varDelta R=\left({S}_{wss}+{S}_p+{S}_m-{S}_s\right)R $$

where *S*_*wss*_ is the local wall shear stress stimulus, *S*_*p*_ is the intravascular pressure stimulus, *S*_*m*_ is the flow carrying hematocrit stimulus, and S_s_ is the natural shrinking tendency of the vessel as a result of the properties of the basal lamina. This natural shrinking tendency is a constant value *k*_*s*_ [44] unless the pressure *P*_*C*_ exerted upon the vessel reaches a critical pressure *P*_*CT*_, at which point the shrinking tendency increases proportionally to the pressure with a rate *k*_*PC*_ to simulate complete vessel collapse, which may partially recover if the stress is relieved [39]:

4 $$ {S}_s={k}_s\kern1em if\kern0.1em {P}_C\le {P}_{CT} $$

5 $$ {S}_S={k}_S+{k}_{pc}\left({P}_c-{P}_{CT}\right)\kern0.5em if\kern0.5em {P}_C>{P}_{CT} $$

In our model, the effect of TEM proximity at a given location is incorporated to provide a protective effect on the neovasculature. Specifically, if a TEM is at an adjacent matrix location to a blood vessel, then the shrinking tendency of the vessel S_s_ iis drastically reduced. Let **1**_TEM_ = 1 denote TEM presence and let **1**_TEM_ = 0 otherwise. The change in vessel radius is then [37]:

6 $$ \varDelta R=\left({S}_{wss}+{S}_p+{S}_m-{S}_s\left(1-{\mathbf{1}}_{\mathrm{TEM}}\right)\right) R\varDelta t $$

### Oxygen transport

Oxygen *σ* is simulated to diffuse with a coefficient *D*_*σ*_ from the location of the vasculature, and is supplied at rates ${\lambda}_{neo}^{\sigma }$ and ${\lambda}_{pre}^{\sigma }$ from the neo- and pre-existing vasculature, respectively. The oxygen is taken up by normal cells with a rate ${\lambda}_{tissue}^{\sigma }$ and by tumor cells with a rate ${\lambda}_{tumor}^{\sigma }$ in the proliferating region and *q*_*σ*_ in the hypoxic region, and decays with rate ${\lambda}_{\mathrm{V}}^{\sigma }$ in the necrotic region. The formulation is [37]:

7 $$ 0=\nabla \cdot \left({D}_{\sigma}\nabla \sigma \right)-{\lambda}^{\sigma}\left(\sigma \right)\sigma +{\lambda}_{ev}^{\sigma}\left(\mathrm{x},t,{1}_{vessel},p,\sigma, h\right) $$

8 $$ {\lambda}^{\sigma }=\left\{\begin{array}{cc}{\lambda}_{tissue}^{\sigma }& \mathrm{outside}\kern0.5em \Omega \\ {}{\lambda}_{tumor}^{\sigma }& \mathrm{in}\kern0.5em {\Omega}_P\\ {}{q}_{\sigma}\left(\sigma \right)& \mathrm{in}\kern0.5em {\Omega}_H\\ {}{\lambda}_N^{\sigma }& \mathrm{in}\kern0.5em {\Omega}_N\end{array}\right. $$

where **x** is position in space, *t* is time, **1**_*vessel*_ is the characteristic function for the vasculature (equals 1 at vessel locations and 0 otherwise), *p* is the tumor (solid) pressure, and *h* is the hematocrit in the vascular network related to oxygen extravasation (following [37]). The extravasation is modulated by the extravascular interstitial pressure *p*_*i*_ scaled by the effective pressure *p*_e_, with ${k}_{P_i}$ being the weight of the convective transport component of small molecules [45]:

9 $$ {\lambda}_{ev}^{\sigma }={\overline{\lambda}}_{ev}^{\sigma }{1}_{vessel}\left(\mathrm{x},t\right){\left(\frac{h}{{\overline{H}}_D}-{\overline{h}}_{\mathrm{min}}\right)}^{+}\left(1-{k}_{Pi}\frac{p_i}{P_e}\right)\left(1-\sigma \right) $$

where ${\overline{\lambda}}_{ev}^{\sigma }$ is the constant transfer rate from both pre-existing and tumor-induced vessels. Constants $\overline{H_D}$ and $\overline{h_{\mathrm{min}}}$ respectively represent normal and minimum blood hematocrit required for oxygen extravasation. The oxygen values are normalized with respect to the vasculature, and hence range from 0 to 1.

### Macrophages

Following [11, 36], monocytes are simulated to extravasate from the vasculature in proportion to the local concentration of macrophage chemoattractants (e.g., pro-angiogenic factors produced by hypoxic tumor tissue), and to preferentially migrate towards tissue regions (e.g., hypoxic tissue) along the increasing gradient of these chemoattractants. Monocytes undergo polarization into M1 or M2 subtypes in the vicinity of the tumor microenvironment based on the concentration of cytokines released by proliferating and hypoxic tumor cells (see Table 3 below). Monocytes and macrophages are simulated as discrete entities using a cellular automaton algorithm.

Since the number of cancer cells is a function of tumor size, one can estimate that a 1mm^3^ tumor lesion may contain up to 3 × 10^6^ cells [46], with about 10% of these cells being macrophages. As in [11], we conservatively assume that the number of macrophages recruited to the lesion is ~25% of that observed in vivo (2.78 × 10^4^ macrophages/mm^3^).

M1 macrophages were simulated to penetrate deeper than the M2 subtypes into the tumor lesion to replicate this effect observed in experiments [36]. This was modeled as a concentric field of value 1 at the tumor center and value 0 at the tumor boundary, selectively biasing the M1 movement based on distance to the center of the lesion.

#### Effects on tumor growth

The effects of macrophage variants M1 and M2 are quantified by their secretion of nitric oxide and tumor growth factors, respectively. This is simulated by the M2 subtype favoring tumor growth by lowering the threshold for tissue to become hypoxic while the M1 subtype counters this effect by secreting NO, which results in tumor tissue death. Their effects *λ*_*M*1_ and *λ*_*M*2_ are incorporated into the proliferation term as follows:

10 $$ {\lambda}_p=\left\{\begin{array}{l} non\kern0.5em tumoral:\\ {}{\varOmega}_P\\ {}{\Omega}_H\\ {}{\Omega}_N\end{array}\right.{\displaystyle \begin{array}{c}\kern17em 0\\ {}\left({\lambda}_M+{\lambda}_{M2}\right)\sigma -\left({\lambda}_A+{\lambda}_{M1}\right)\\ {}\kern5em {\lambda}_{M2}\kern0.5em \sigma -\left({\lambda}_A+{\lambda}_{M1}\right)\\ {}\kern16.5em -{G}_N\end{array}} $$

where *λ*_*M*_ is the tumor native mitosis rate, *σ* is the local oxygen concentration calculated via Eq. 3 and *λ*_*A*_ is the apoptosis rate due to natural tumor cell death. The non-dimensionalized rate of cell degradation in the necrotic region is *G*_*N*_, which assumes that cellular necrotic debris is constantly degraded and the associated fluid is removed. The M1 cytotoxicity is modeled to affect both proliferating (cycling) and hypoxic (quiescent) tissue, since the death mechanism is assumed to be cell-cycle independent.

The cytotoxic effect *λ*_*M*1_ of the M1 subtype is simulated to affect tissue proportional to the release rate *λ*_*NO*_ of nitric oxide in the immediate vicinity of the macrophage (**1**_***M*1**_), since nitric oxide has a short half-life in vivo with limited diffusion distance.

11 $$ {\lambda}_{M1}={\lambda}_{NO}{\mathbf{1}}_{\mathrm{M}1} $$

In addition to inhibiting tumor death, the presence of the M2 growth factor has a positive effect on the proliferating region as follows:

12 $$ \frac{d{\lambda}_{M2}}{dt}={\lambda}_FF\left(1-\left({\lambda}_M+{\lambda}_{M2}\right)\right) $$

where *λ*_*M*2_ is the proliferation rate due to the concentration *F* of the diffusible M2 growth factor, which adds to the native proliferation rate *λ*_*M*_, and *λ*_*F*_ is the effect of the M2 growth factor on the proliferation. The proliferation effect due to *λ*_*M*2_ decreases as the net proliferation (*λ*_*M*_ + *λ*_*M*2_) approaches a maximum value of 1 day^−1^. M2 macrophages can also stimulate the quiescent (hypoxic) tumor cells to proliferate, albeit at lower rates than well perfused tissue (see below).The tumor growth factor concentration *F* secreted by the M2 macrophages achieves a transient local lowering of the viable oxygen threshold – the oxygen level below which tumor cells die – as follows:

13 $$ \frac{dQ_{OL}}{dt}={\lambda}_{OL}\cdot \left(1-F\right)\cdot \left({\overline{Q}}_{OL}-{Q}_{OL, current}\right)-{\lambda}_{OT}\cdot F\cdot \left({Q}_{OL, current}-{Q}_{OL,\min}\right)\cdot $$

where *Q*_*OL*_ is the effective quiescence oxygen level, *λ*_*OL*_ is the recovery rate of *Q*_*OL*_ to the standard quiescence oxygen level ${\overline{Q}}_{OL}$, *Q*_*OL*, *current*_ is the current quiescence oxygen level, *F* is the local concentration of M2 growth factor (ranges from 0 to 1, dimensionless units), *λ*_*OT*_ is the M2 growth factor effect rate on the lowering of the viable oxygen threshold, and *Q*_*OL min*_ is the lower bound of the quiescence oxygen level. The effective oxygen level is set to ${\overline{Q}}_{OL}$ if it exceeds ${\overline{Q}}_{OL}$, and to *Q*_*OL min*_ if it is less than *Q*_*OL min*_.

#### Differentiation

Given the increased ratio of M2/M1 macrophages typical of tumor lesions, the role of TEM-produced IL-10 on the ratio of M2/M1 macrophage subtypes is modeled. A target range of 0.32–5.23 for this ratio was used, to match in vitro data for metastatic tumors in the liver [47].

As monocyte precursors *Mϕ* extravasate from the vasculature in the tumor region, they come into contact with cytokines diffusing from the tumor interior and the vasculature that influence their differentiation. The concentration of factors, analogous to interleukins and others that encourage differentiation of given subtypes, influences the respective differentiation rate *R*_*i*_ dependent on the size of the interval that a randomly generated number may fall into. Thus, the differentiation probabilities depend on the concentration of factors:

14 $$ {\displaystyle \begin{array}{c}{R}_{M1}\propto {k}_{M1}\cdot {C}_{M1f}\\ {}{R}_{M2}\propto {k}_{M2}\cdot \left({C}_{M2f}+{k}_{T2M2}\cdot {C}_{IL-10}\right)\\ {}{R}_{TEM}\propto \left({k}_{T2}\cdot {C}_{T2f}+{k}_{Ang2}{C}_{Ang2}\right)\end{array}} $$

where *k*_*M*1_, *k*_*M*2_, *k*_*T*2_ are intensity coefficients tuned to reflect the relative prevalence of M1 or M2 differentiating monocytes and Tie2 expressing macrophages infiltrating the tumor, *C*_*M*1*f*_, *C*_*M*2*f*_, *C*_*IL* − 10_, *C*_*T*2*f*_ and *C*_*Ang*2_ are local concentrations of cytokines and other factors favorable to M1, M2, or TEM differentiation released by the viable (proliferating or hypoxic) tumor regions, and *k*_*Ang*2_ and *k*_*T*2*M*2_ are intensity coefficients to tune the effect of Ang2 and Il-10 favoring M2 differentiation, respectively.

#### Cytokine production and diffusion

Assuming steady-state conditions, the overall mass balance for a particular cytokine concentration *C* (dimensionless units) produced by the viable (proliferating and hypoxic) tumor regions is [48]:

15 $$ 0=\nabla \cdot \left({D}_C\nabla C\right)+{\overline{\lambda}}_{production}^C\left(1-C\right){\mathbf{1}}_{\Omega}-{\overline{\lambda}}_{circulation}^C{\mathbf{1}}_{vessel}-{\overline{\lambda}}_{decay}^CC $$

where *D*_*C*_ is diffusivity and ${\overline{\lambda}}_{production}^C$, ${\overline{\lambda}}_{circulation}^C$, and ${\overline{\lambda}}_{decay}^C$ are the (constant) non-dimensional rates of cytokine production, wash-out via circulation, and decay, respectively. The values for concentration range from 0 to 1.

For all the diffusion equations, as well as the pressure and angiogenic factors, zero Neumann conditions are taken at the boundaries [37].

#### Movement

Monocytes and M1 and M2 macrophages migrate through the interstitium guided by gradients of oxygen, pressure, and chemoattractants. Movement in one of four directions along the computational grid is determined semi-stochastically, similar to their differentiation described earlier. The probability of movement in the x + 1 direction is as follows:

16 $$ {P}_{x+1}=\left({M}_O\cdot \varDelta {O}_{x+1}+{M}_P\cdot \varDelta {P}_{x+1}+{M}_C\cdot \varDelta {Chemo}_{x+1}\right) $$

where *M*_*O*_, *M*_*P*_ and *M*_*C*_ are intensity coefficients for the influence of oxygen concentration, pressure, and chemoattractant on macrophage movement, and *∆O*_*x* + 1_, *∆P*_*x* + 1_ and *∆Chemo*_*x* + 1_ are the difference in concentration of the factor of interest from the current point to the direction in question. The same calculations are made for the remaining three directions in the 2D Cartesian grid. Each probability is divided by the total sum of the four probabilities, and intervals are then defined proportional to the respective magnitude of these scaled probabilities. A random number ranging from 0 to 1 is generated, and movement in a specific direction is determined based on which interval the number falls into. If no interval qualifies, the macrophage remains in place. Although a macrophage is allowed to share the same location on the grid as a vessel, only one monocyte or macrophage can occupy a grid location.

The method of movement for the TEM is also semi-stochastic, but relies upon a different chemoattractant, namely Ang2 gradients secreted by the neovasculature. The probability is modeled as follows:

17 $$ {P}_{x+1}={M}_{Ang2}\cdot \varDelta Ang{2}_{x+1} $$

where *M*_*Ang*2_ is the intensity coefficient tuned to scale the response of TEMs to the angiopoetin2 concentration gradient in each direction.

#### Parameter calibration

The values of macrophage-associated parameters are defined in Table 2. These parameters were set to values in the literature or calibrated so that the simulated tumor growth would match experimentally measured values in the literature. The cytokine characteristics are summarized in Table 3, based on prior work that classified protein diffusivity based on molecular weight [48]. The wash-out rate into the vasculature, decay rate, diffusivity, and production rate are shown for each cytokine in Table 4. The concentration of IL-10 in pg/mL is calculated by treating each pixel in the spatial model as a 3-dimensional voxel. Thus, the final concentrations for IL-10 in simulations with the TEM subtype present are within previously observed values of 5.6–37 pg/mL for breast cancers of various TNM stages [49].

**Table 2:** Description of macrophage-associated parameters

Parameter	Description	Value	Reference
*Physiological Parameters*			
% of macrophages per tumor total cells		10%	Calibrated to match [11]
TEM-driven tumor neovasculature increase		4-fold	[12]
TEM portion of differentiated macrophages		55–70%	[13]
M2/M1 ratio in highly metastatic tumors		2.06	[47]
M2/M1 ratio in moderately metastatic tumors		0.77	[47]
*Vessel Radius-Associated Parameters Related to TEM Effects*			
*k*_*s*_	Natural shrinking tendency of vessel radius	2.24	[39]
*k*_*PC*_	Response rate of radius to tumor pressure	0.76	[39]
*Macrophage-Associated Parameters Related to Tumor Growth*			
*λ*_*M*_	Tumor native proliferation rate (day^−1^)	0.5	Calibrated to match [11]
*λ*_*OL*_	Recovery rate of quiescent oxygen level	0.05(^*^)	Calibrated to match [36]
*λ*_*OT*_	M2 induced lowering viable O_2_ threshold rate	200 /s	Calibrated to match [36]
*λ*_*rec*_	Recovery rate of *λ*_*M*2_to zero	0.1(^*^)	Calibrated to match [36]
*λ*_*F*_	M2 induced proliferation rate	1000 /s	Calibrated to match [36]
*λ*_*NO*_	M1 nitric oxide induced death rate	3 /s	Calibrated to match [8]
*G*_*N*_	Cell degradation rate in the necrotic region	0.3(^*^)	Calibrated to match [11]
*Macrophage Differentiation Scaling Coefficients*			
*k*_*M*1_	Differentiation of M1 macrophage	20	[36]
*k*_*M*2_	Differentiation of M2 macrophage	20	[36]
*k*_*T*2_	Differentiation of TEM	8.21	[13]
*k*_*Ang*2_	Effect of Ang2 on TEM differentiation	0.95	Calibrated to match [1, 13]
*k*_*T*2*M*2_	Effect of IL-10 on M2 differentiation	0.006	Calibrated to match [47]
*Macrophage Movement Scaling Coefficients*			
*M*_*O*_	Effect of oxygen on macrophage movement	1000	[11]
*M*_*P*_	Effect of oxygen on macrophage movement	350	[11]
*M*_*C*_	Chemotactic macrophage movement	500	[11]
*M*_*Ang*2_	Effect of Ang2 on TEM movement	1000	Calibrated to match [16]

(^*^) Value is non-dimensionalized by the diffusivity of oxygen [68] (1 × 10^−5^ cm^2^ s^−1^)

**Table 3:** Characteristics of the macrophage-associated cytokines used in this study. The M1f characteristics were chosen to be similar to IL-6, while the M2f characteristics were chosen similar to IL-10

Cytokine	Function	Source	MW (Da)	Diffusivity (as fraction of TAF diffusivity)
M1f	M1 differentiation	Proliferating & hypoxic tumor cells	21,000	1
M2f	M2 differentiation	Proliferating & hypoxic tumor cells	18,606	3.7606
IL-10	TEM-eluted factor	TEM	18,606	3.7606
T2f	TEM differentiation	Proliferating & hypoxic tumor cells	60,179	1
Ang2	TEM chemoattractant	Neovasculature	~70,000	0.26591

**Table 4:** Macrophage-associated cytokine parameters based on proteomic analysis in Frieboes et al. [48]

Parameter	Function	Value
${\overline{\lambda}}_{circulation}^C$	Wash-out rate into vasculature	0.006 (^*^)
${\overline{\lambda}}_{decay}^C$	Decay rate	0.001 (^*^)
*D*_*M*1*f*_	Diffusivity for *M1f*	0.005 (^*^)
*D*_*M*2*f*_	Diffusivity for *M2f*	0.01880 (^*^)
*D*_*IL* − 10_	Diffusivity for *IL-10*	0.01880 (^*^)
*D*_*T*2*f*_	Diffusivity for *T2f*	0.005 (^*^)
*D*_*Ang*2_	Diffusivity for *Ang2*	0.00133 (^*^)
${\overline{\lambda}}_{production}^{M1f}$	production rate of *M1f*	1.0 (^**^)
${\overline{\lambda}}_{production}^{M2f}$	production rate of *M2f*	1.0 (^**^)
${\overline{\lambda}}_{production}^{IL-10}$	production rate of *IL-10*	1.0 (^**^)
${\overline{\lambda}}_{production}^{T2f}$	production rate of *T2f*	1.0 (^**^)
${\overline{\lambda}}_{production}^{Ang2}$	production rate of *Ang2*	1.0 (^**^)

The same wash-out and decay rates apply to all cytokines, generically denoted by *C*. (^*^) Value is non-dimensionalized by the diffusivity of oxygen [68] (1 × 10^−5^ cm^2^ s^−1^). (^**^) Value is rescaled by the production rate of VEGF-A (VEGF -165) protein, representing a typical TAF molecule

### Numerical implementation

Details of the numerical implementation are described in [39] and references therein. Briefly, to solve for the tumor oncotic pressure and the diffusible elements (oxygen, growth factors, cytokines, as well as tumor angiogenic factors and matrix-degrading enzymes included in the angiogenesis model), the corresponding equations are discretized in space using centered finite difference approximations and the backward Euler time-stepping algorithm. The discretized equations are solved using a nonlinear adaptive Gauss–Seidel iterative method [50, 51]. A ghost cell method is used to implement the tumor pressure jump condition at the tumor-host interface [51]. This system of equations is iteratively solved together for the tumor oncotic pressure and the concentration of diffusible elements (as well as interstitial fluid pressure and blood vessel pressure in the angiogenesis component [39]) to steady state at each timestep, i.e., the equations are discretized implicitly in time. The level set method is used to update the tumor viable/necrotic region and the interfaces between the tumor–host and tumor viable–necrotic tissue regions. In the angiogenesis component, the vessel radii are discretized explicitly, and the hematocrit level is calculated every few iterations. This hematocrit is modulated by the blood flow and influences the extravasation of oxygen from the vasculature. Further details regarding the numerical implementation are in [37] and references therein.

## Results

### Combinations of macrophage phenotypes

The tumor, vascular, and macrophage parameters were first calibrated as described in **Methods**. The single and combined effects of the three macrophage types on tumor growth were then evaluated in various cases, as follows:

**Table:** 

Case 1	Case 2	Case 3	Case 4	Case 5	Case 6	Case 7	Case 8
M1	M1	M1		M1			(None)
M2	M2		M2		M2		
TEM		TEM	TEM			TEM	

A case to match in vivo macrophage ratios [13] was first run, with all three macrophage subtypes present (Case 1). The other cases then examined the tumoral response to the other possible population combinations. Each case was observed over a simulated 13-day timespan, and the results were compared with existing experimental data. The number of monocyte precursors infiltrating the tumor microenvironment changed dynamically in time based on the density of the vasculature and the concentration of tumor angiogenic factors (e.g., VEGF) released by the hypoxic tumor tissue. In order to maintain consistent differentiation probabilities for all the cases, monocytes differentiating into a subtype absent in a particular scenario were rendered to have no effect. In this manner, the pool of monocyte precursors available to differentiate into the effective subtypes remained consistent across the cases by making it dependent only on the changing tumor conditions (size, vascularization, hypoxic tissue, etc.).

### Tumor growth influenced by macrophage phenotypes

**Fig. 1:**
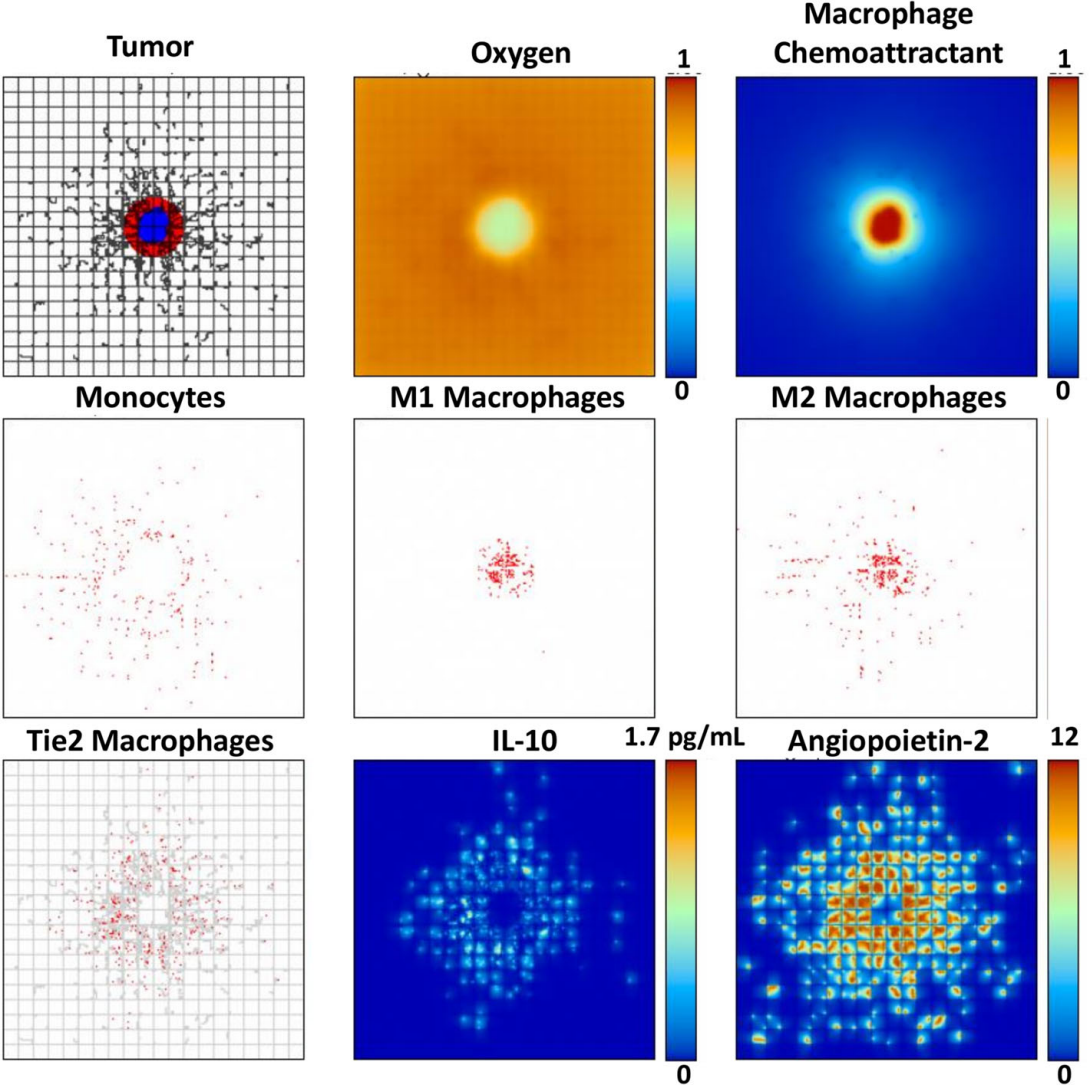
Tumor growth by 13 days with TEM and M1 and M2 macrophage subtypes present. Top left: Tumor with proliferating regions in red and quiescent in blue. Vessels here are shown as black lines, with preexisting vessels comprising the grid space and angiogenic vessels depicted as irregular offshoots. Top center: Oxygen gradient, showing hypoxic conditions in the tumor interior. Top right: Macrophage chemoattractant, (e.g., tumor angiogenesis factors) secreted by the hypoxic tissue of the tumor. Middle left: Monocytes extravasated from the vasculature. Middle center: M1 macrophages shown in red. Middle right: M2 macrophages shown in red. Bottom l eft: TEMs shown in red with vasculature superimposed in gray. The Angiopoietin-2 secreted by the vasculature has caused the TEMs to cluster around the neoangiogenic sprouts. Bottom center: IL-10 secreted by the TEM, which favors monocyte differentiation into M2. Bottom right: Angiopoietin-2 secreted by the neovasculature, which attracts the TEMs to accumulate by the vessels. Each panel represents 4 mm^2^

The tumor growth along with the associated vascular development, oxygen, macrophage infiltration, and key secreted factors, at 13 days post inception for Case 1 are shown in Fig. 1. By this time the tumor has developed a proliferating region, mostly on the periphery, surrounding a hypoxic interior. The extravasation of monocytes and subsequent macrophage differentiation into M1, M2, or TEM phenotypes is triggered by the release of macrophage chemoattractants (e.g., TAF) from the tumor hypoxic tissue, as well as by the concentration of Ang2 secreted by the neovasculature, favoring monocyte differentiation into TEM (Eq. 14). The monocyte infiltration began when the lesion reached 200 μm in diameter (7.35 days post inception), at the onset of hypoxia. The M1 subtypes are mostly concentrated within the tumor lesion, while the M2 subtypes are more spread in the immediate periphery as a consequence of the monocyte contact with the TEM-eluted factor. Following the gradient of Ang2, the TEMs preferentially cluster around angiogenic vessels, where they hinder vessel shrinking and collapse due to increased pressure of the growing tumor, as expressed in Eqs. 4–6. This vessel relief enables the tumor to have increased access to oxygen and nutrients. Meanwhile, the TEM secretion of IL-10 favors monocyte differentiation into the M2 subtype, as described by Eq. 14.

**Fig. 2:**
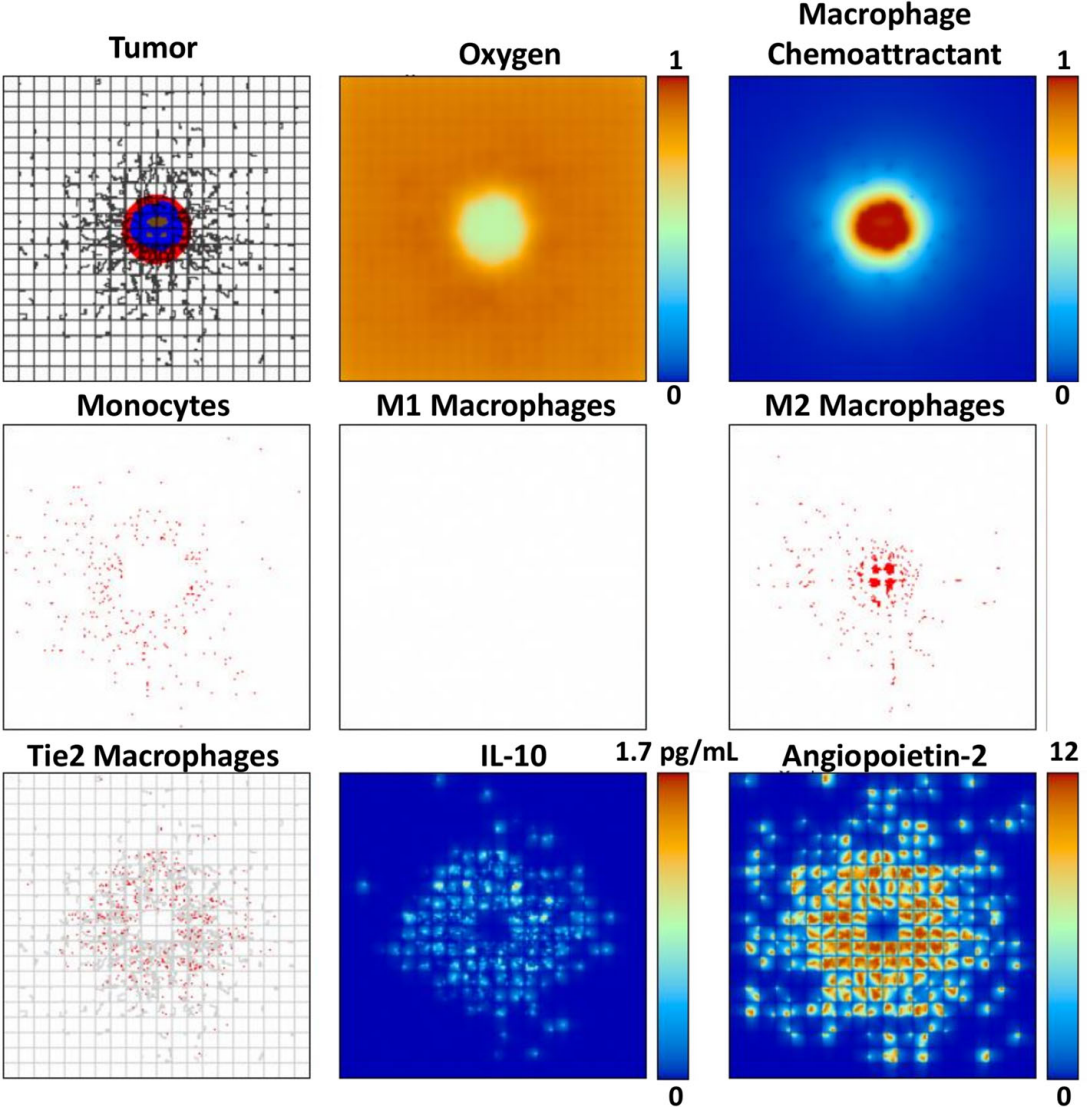
Tumor growth by 13 days with both TEM and M2 macrophage subtypes present (same description of panels as in Fig. 1). The M2 macrophages penetrate into the tumor following the gradient of macrophage chemoattractants, with their distribution more scattered than the M1 in the vicinity of the tumor due to the monocyte contact with the TEM-eluted IL-10. The tumor is substantially larger than in Fig. 1, and has more hypoxia

**Fig. 3:**
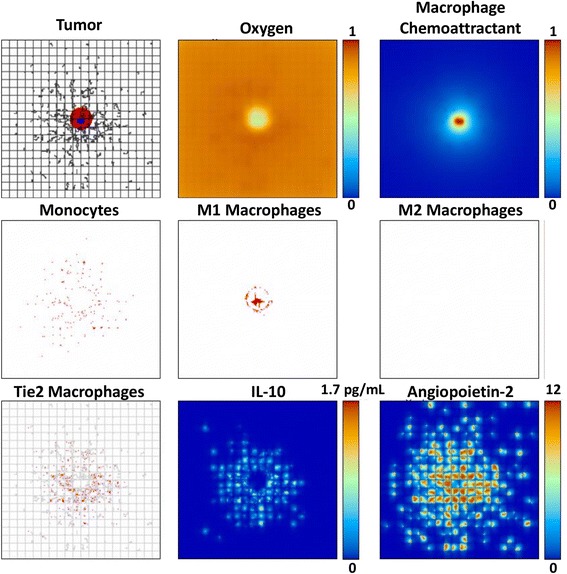
Tumor growth by 13 days with both TEM and M1 macrophage subtypes present (same description of panels as in Fig. 1). The M1 macrophages penetrate into the tumor following the gradient of macrophage chemoattractants while the TEM remain close to the neovascular network. The tumor is significantly smaller than in Fig. 1, and has less hypoxia

**Fig. 4:**
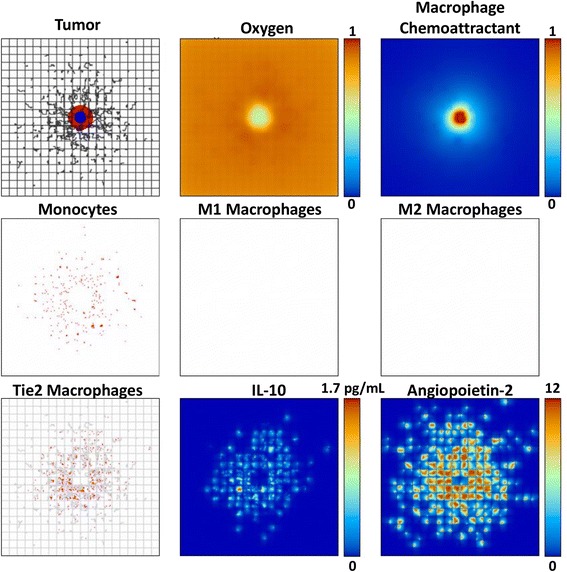
Tumor growth by 13 days with only the TEM subtype present (same description of panels as in Fig. 1). In this case, the size of the tumor and its associated hypoxia lie in between the TEM/M2 and TEM/M1 cases

The tumor and associated macrophage parameters on day 13 when the M1 subtype is removed from the system (Case 4), leaving only the possibility of M2 or TEM phenotypes, are shown in Fig. 2. Unhindered by the M1 cytotoxic effect, in this case the tumor is able to grow larger than the case when all subtypes are present (Fig. 1). In contrast to this situation, Fig. 3 illustrates a substantially smaller tumor when the M2 subtype is removed and only the M1 and TEM subtypes are present (Case 2). This case is not as favorable for tumor growth as might be expected, as the TEM pro-tumor effects are less pronounced and on a longer time scale than the M1 cytotoxicity. This is confirmed when both M1 and M2 phenotypes are removed and only the TEM are present (Case 7), as shown in Fig. 4.

### Ratio of macrophage phenotypes

**Fig. 5:**
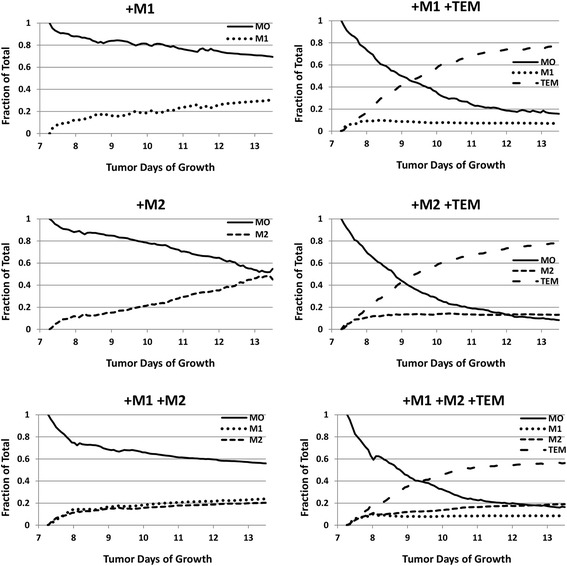
Time evolution of the macrophage sub-populations depending on the type of the sub-populations. Left column (from top to bottom): M1-only, M2-only, and M1/M2 cases. Right column: as in left column plus TEM. MO: monocytes

As the tumor grows in time, the number of macrophages changes dynamically based on the concentration of the relevant cytokines, which, depending on the cytokine, is coupled in the model between the macrophages, the tumor and the vasculature, as described in the Methods. The proportion of different macrophage types during the tumor growth is shown in Fig. 5. When present, Tie2 expressing macrophages differentiating from the monocyte precursors became in all cases the majority subset in the tumor environment (77% for M1/TEM, 78% for M2/TEM, and 56% for M1/M2/TEM), matching in vivo data [13]. Without TEM, the M1:M2 ratio stabilized at 1.2:1.0. This ratio is half the median ratio for highly metastatic tumors, and within the normal range for more benign tumor populations [47]. The secretion of IL-10 when TEM were present shifted the M1:M2 ratio as high as 1.0:2.2 - a ratio more consistent with highly metastatic tumors [47].

### Vascular development influenced by macrophage phenotypes

**Fig. 6:**
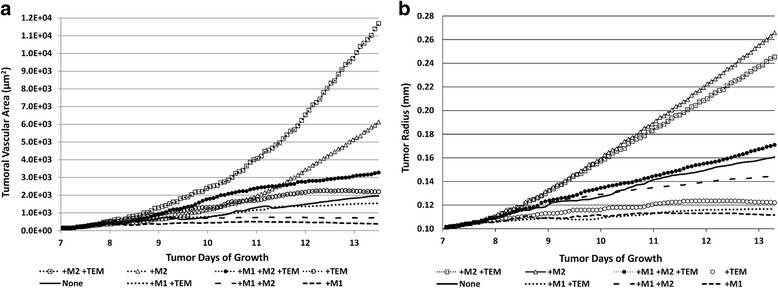
Time evolution of the tumor vasculature and tissue as a function of macrophage sub-populations. **a** Tumor vascular surface area (μm^2^) calculated using actual capillary radii values influenced by pressure and TEM effects. **b** Tumor radius (mm). None: no macrophages present

Due to the protective clustering around angiogenic vasculature, cases with the TEM subtype had notably greater vascular development compared to the TEM-absent cases (Fig. 6a), with two exceptions. The M1/TEM model elicited less vascular development than that of the macrophage-absent scenario, due to the M1 tumoricidal effect curtailing the tumor growth, while the M2-only case achieved the largest intratumoral vascularity other than the M2/TEM, which is due to the promotion of tumor growth by the M2 subtype. Comparing the case of M1/M2/TEM to M1/M2, in which TEM is respectively present and ablated, a 4.6-fold increase in tumoral vasculature is observed by day 13. This is greater than the four-fold increase found by De Palma et al. in an analogous study in vivo [12]. However, this may be expected as the macrophages in the simulation continue to enter the environment, replenishing the macrophages that die, whereas the in vivo study used a single bolus dose which was not replaced in the TEM-absent mice. For comparison, the M2/TEM case had 1.9-fold more vasculature than the M2-only, while the M1/TEM case had 4.9-fold more vasculature than the M1-only scenario.

### Analysis of tumor growth over time

The TEM subtype protective effect on angiogenic vessels and promotion of M2 differentiation in concert had a noticeable effect on the tumor progression (Fig. 6b). While all cases achieved a size that at least transiently rendered the interior portions hypoxic, only those with the TEM or M2 subtypes were able to achieve continued growth through the end of the simulation. The case with all three subtypes yielded 18% increase in tumor radius over the TEM-ablated model by day 13 of the simulation. The M2 scenario exhibited the largest growth, despite the development of hypoxic and necrotic regions within the tumor lesion. All TEM-ablated cases without M2 showed a plateau in growth around Day 11. The presence of the M2 enabled the tumor to overcome the absence of the TEM and mitigate the tumoricidal effect of the M1, as shown by the case with M2-only eliciting the second-highest overall growth and the case of M1/M2 showing continued growth, respectively. The case with M1-only showed a more dramatic plateauing of growth, consistent with findings of M1-only in vivo [8].

**Fig. 7:**
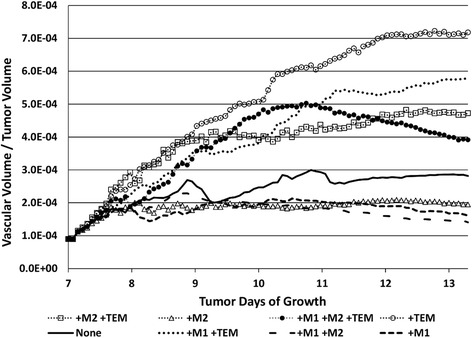
Size of tumor vasculature and tumor on day 13 post inception as a function of macrophage sub-populations. **a** Tumor vascular surface area (μm^2^) assuming capillary sizes of 10 μm diameter. **b** Tumor radius (mm). None: no macrophages present

The extent of vascularization and the tumor growth by day 13 are summarized in Fig. 7. While the M2/TEM case had twice the vascular area of the M2-only case, the latter exhibited the largest growth. This indicates that the M2 growth-promoting effect is more potent due to a tumor proliferative response that is more rapid than the vasculature-stabilizing effect of the TEM. This is supported by the observation that the tumor growth with M1/M2 was 24% larger than the M1/TEM case, even though the vascular development with M1/M2 was only 47% that of the M1/TEM case.

### Tumor growth dependent on macrophage-influenced vascularization

**Fig. 8:**
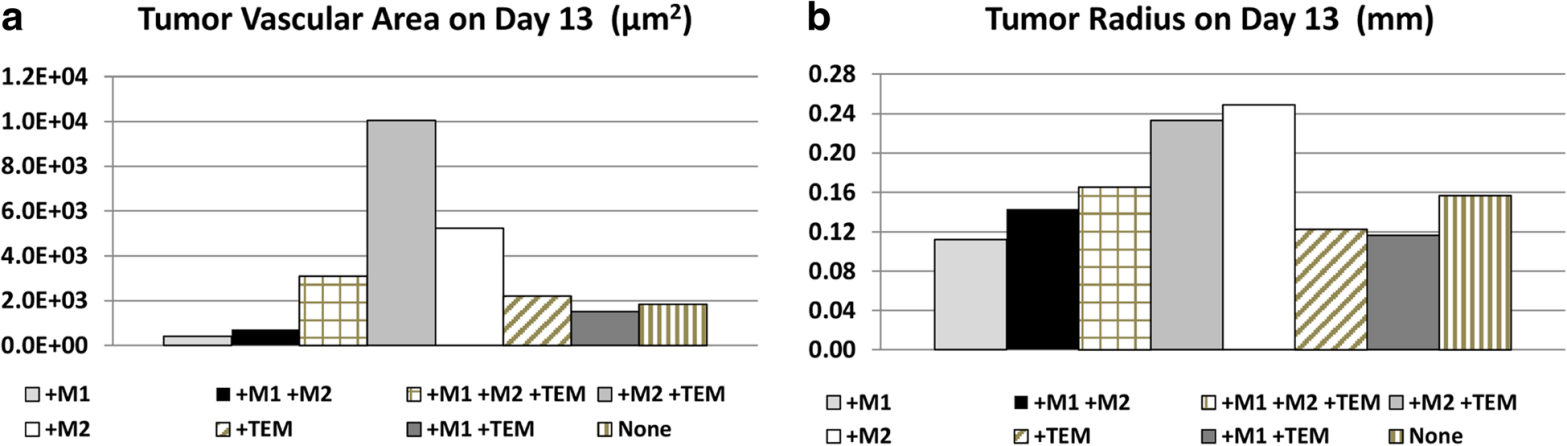
Time evolution of ratio of vascular volume to tumor volume as a function of macrophage sub-populations present. None: no macrophages present

The ratio of tumor vascular volume to tumor tissue volume evolving in time (Fig. 8) shows, as expected, that the cases with TEM present had the highest ratios by 13 d, with the TEM-only case having the highest (7.2 × 10^−4^). The lowest ratio was for the M1/M2 case (1.5 × 10^−4^). The presence of the M2 subtype lowers these ratios because the stimulation of proliferation by M2 macrophages is faster than vascular development driven by the TEM. Interestingly, the ratio for the case with M1/M2/TEM first peaks on day 10 at 5 × 10^−4^ and then declines to 3.9 × 10^−4^ by day 13, suggesting a sequence of initially higher vascular development followed by increasing tumor volume. The ratio for the M1/M2 scenario also slightly declines after peaking at 2.3 × 10^−4^ on day 8, implying a progressively increasing tumor volume, while the M1-only case declines to 1.6 × 10^−4^ by day 13 due to the combination of shrinking vasculature as well as tumor volume. All the other cases appear to plateau to constant ratios (M2 to 1.9 × 10^−4^, M2/TEM to 2.7 × 10^−4^, M1/TEM to 5.8 × 10^−4^, and with none to 2.8 × 10^−4^), which are values consistent with previous modeling work evaluating tumor growth as a function of vascularization [52].

## Discussion

This study employed mathematical modeling to explore the tumor-promoting and tumor-inhibiting roles of three major TAM subtypes, namely, the M1 cytotoxic, the M2 tumor growth-promoting, and the TEM vasculature-stabilizing phenotypes. Model parameters were calibrated to biologically-relevant values. A small, hypo-vascularized lesion was simulated growing in a highly vascularized microenvironment, such as in the lung or the liver, and the tumor growth, vasculature remodeling, and the macrophage activity were coupled. The results show that all cases with the M2 phenotype led to larger tumor growth regardless of TEM presence or not. The implication is that immunotherapeutic strategies leading to ablation of the TEM phenotype may provide limited benefit to restrain this growth when the M2 macrophages represent a sizeable population. On the other hand, the vasculature-stabilizing effect of the TEM could perhaps be leveraged to achieve more homogeneous chemotherapy penetration into the tumor tissue, which is a notorious challenge presented by hypo-vascularized tumor lesions [11]. This suggests the intriguing possibility that an immunotherapy that promotes monocyte differentiation to TEM during chemotherapy followed by therapy that ablates both TEM and M2 phenotypes during intervening cycles could represent a more optimal strategy.

Given the ability of tumors to educate infiltrating macrophages to a tumorigenic subtype, methods of countering this may prevent tumors from harnessing the body’s most potent effectors of tissue remodeling as has been previously suggested [53]. According to this paradigm, therapies which primarily inhibit monocytes and macrophages that infiltrate the tumor environment would not be ideal as they would fail to utilize the inherent tumoricidal activity of M1 macrophages. However, the modeling results indicate that there is a wide separation of time scales in the actions of the M1, M2 and TEM macrophages. In particular, the TEM pro-tumor effects are less pronounced and on a longer time scale (days/weeks) than the M1 tumor-inhibiting effects (minutes/day). Thus, a more finessed approach to influence monocyte differentiation that takes into account the state of the tumor (e.g., under chemotherapy or not) may be desirable.

Since the TEM phenotype is also implicated in the facilitation of cancer metastasis by degrading the ECM and guiding metastatic cells to the vasculature (e.g., as observed with breast cancer [54]), selective and timely blockade of this subtype from the tumor microenvironment may be essential to successfully halt and reverse the progression of cancer in patients [13]. The invasiveness-promoting action of the TEM subtype may in part explain the pitfalls seen in the use of VEGF inhibitors [55]. Due to the tendency, in some cases, of inhibited tumoral blood supply to lead to increased tumor spread through fragmentation and migration of tumor cells, preventing the vasculature from growing towards the tumor could force a more malignant and metastatic phenotype, as has been observed experimentally [56–59], clinically [56], and predicted by mathematical modeling [60–62]. Given that TEMs differentiate from a monocyte precursor distinct from the M1 and M2 subtypes, the possibility of TEM-specific therapies may present a viable approach to influence tumor behavior [63].

Future work will explore the interaction of tumor and macrophage effects during treatment. Therapy could be delivered systemically as free drug or encapsulated in nanovectors, as previously simulated [64–66], or its delivery to the tumor site could be targeted by TAM uptake and release [11, 36]. Pharmaceutical ablation of tumor-promoting subtypes while supporting tumor-inhibiting phenotypes could provide nuanced therapeutic options. The combination of various modalities could be explored via the modeling framework presented herein, as such options may be difficult to evaluate solely through experimental observation. To the extent that current oncology treatment options, such as chemotherapy and immunotherapy, favor one or more macrophage subtypes over other ones, the results of this study would be applicable in projecting how the resulting proportion of subtypes may influence the tumor progression and ultimately the response to the therapy.

As this work represents an initial implementation, incorporation of additional biological details may provide enhanced modeling capability. It was recently reported that Programmed Cell Death Protein 1 (PD-1) expression by tumor-associated macrophages inhibits tumor immunity [67]. It was observed that almost all of the PD-1-expressing TAMs were of the M2 subtype, and that their proportion increased significantly with the disease stage. The simulation of PD-1 expression by TAMs in this model would be expected to facilitate tumor survival by potentiating the tumor-promoting effect of the M2 macrophages, which would also indirectly influence the TEM activity. Further, the addition of a stromal component dependent on ECM production and remodeling would affect the transport of oxygen and nutrients, as well as the diffusivity of tumor- and macrophage-released cytokines, which in turn would affect the TAM response. A denser ECM, for example, could impede the diffusive transport while also potentially limiting the tumor’s growth pattern. The modeling of primary instead of metastatic lesions could be represented by changing the host vasculature pattern and macrophage subtype populations to match that of specific organs, as well as simulating potentially larger tumor masses. Such biological details would be required to adapt the model to different cancer types, for which the defining characteristics would include particular tumor growth, vascular, stromal, and immune conditions to be calibrated with the associated model parameters. With input of patient tumor-specific information, such as size, vascularization, and macrophage presence, this framework may in the longer term serve to determine optimal therapy regimens leveraging the body’s immune response to cancerous lesions.

## Conclusions

This work provides a modeling platform for system analysis of the potent and varied effects of the macrophage activation spectrum on the tumor microenvironment, with the goal to complement current cancer therapy design. The results of this initial implementation show that TEM ablation as an immunotherapeutic strategy may fail to restrain growth when the M2 represents a sizeable population. Further, an approach that leverages the vasculature-stabilizing effect of the TEM during chemotherapy may be desirable.

## Abbreviations

2D: Two-dimensional
IL-10: Interleukin 10
IL-6: Interleukin 6
MMP-9: Matrix metallo-proteinase 9
NO: Nitric oxide
TAF: Tumor angiogenic factors
TAM: Tumor-associated macrophages
TEM: Tie2-expressing macrophages
TGF-β1: Tumor growth factor β1
VEGF-A: Vascular endothelial growth factor A

## References

[1] Chanmee T, Ontong P, Konno K, Itano N (2014). Tumor-associated macrophages as major players in the tumor microenvironment. Cancers.

[2] De Palma M, Lewis CE (2013). Macrophage regulation of tumor responses to anticancer therapies. Cancer Cell.

[3] Squadrito ML, De Palma M (2011). Macrophage regulation of tumor angiogenesis: implications for cancer therapy. Mol Asp Med.

[4] Laoui D, Movahedi K, Van Overmeire E, Van den Bossche J, Schouppe E, Mommer C, Nikolaou A, Morias Y, De Baetselier P, Van Ginderachter JA (2011). Tumor-associated macrophages in breast cancer: distinct subsets, distinct functions. The International journal of developmental biology.

[5] Italiani P, Boraschi D (2014). From Monocytes to M1/M2 macrophages: Phenotypical vs. Funct Differ Front Immunol.

[6] Plank MJ, Sleeman BD (2003). Tumour-induced angiogenesis: a review. J Theor Med.

[7] Edin S, Wikberg ML, Dahlin AM, Rutegård J, Öberg Å, Oldenborg P-A, Palmqvist R (2012). The distribution of macrophages with a M1 or M2 phenotype in relation to prognosis and the molecular characteristics of colorectal cancer. PLoS One.

[8] Yuan A, Hsiao Y-J, Chen H-Y, Chen H-W, Ho C-C, Chen Y-Y, Liu Y-C, Hong T-H, Yu S-L, Chen JJW, Yang P-C (2015). Opposite effects of M1 and M2 macrophage subtypes on lung cancer progression. Sci Rep.

[9] Sica A, Mantovani A. Macrophage plasticity and polarization: in vivo veritas. J Clin Invest. 122(3):787–95. doi: 10.1172/JCI59643. 10.1172/JCI59643 PMC3287223 22378047

[10] Murdoch C, Giannoudis A, Lewis CE (2004). Mechanisms regulating the recruitment of macrophages into hypoxic areas of tumors and other ischemic tissues. Blood.

[11] Leonard F, Curtis LT, Yesantharao P, Tanei T, Alexander JF, Wu M, Lowengrub J, Liu X, Ferrari M, Yokoi K, Frieboes HB, Godin B (2016). Enhanced performance of macrophage-encapsulated nanoparticle albumin-bound-paclitaxel in hypo-perfused cancer lesions. Nano.

[12] De Palma M, Venneri MA, Galli R, Sergi LS, Politi LS, Sampaolesi M, Naldini L (2008). Tie2 identifies a hematopoietic lineage of proangiogenic monocytes required for tumor vessel formation and a mesenchymal population of pericyte progenitors. Cancer Cell.

[13] Venneri MA, De Palma M, Ponzoni M, Pucci F, Scielzo C, Zonari E, Mazzieri R, Doglioni C, Naldini L (2007). Identification of proangiogenic TIE2-expressing monocytes (TEMs) in human peripheral blood and cancer. Blood.

[14] Patel AS, Smith A, Nucera S, Biziato D, Saha P, Attia RQ, Humphries J, Mattock K, Grover SP, Lyons OT, Guidotti LG, Siow R, Ivetic A, Egginton S, Waltham M, Naldini L, De Palma M, Modarai B (2013). TIE2-expressing monocytes/macrophages regulate revascularization of the ischemic limb. EMBO Mol Med.

[15] Matsubara T, Kanto T, Kuroda S, Yoshio S, Higashitani K, Kakita N, Miyazaki M, Sakakibara M, Hiramatsu N, Kasahara A, Tomimaru Y, Tomokuni A, Nagano H, Hayashi N, Takehara T (2013). TIE2-expressing monocytes as a diagnostic marker for hepatocellular carcinoma correlates with angiogenesis. Hepatology.

[16] De Palma M, Naldini L (2011). Angiopoietin-2 TIEs up macrophages in tumor angiogenesis. Clin Cancer Res.

[17] Riabov V, Gudima A, Wang N, Mickley A, Orekhov A, Kzhyshkowska J (2014). Role of tumor associated macrophages in tumor angiogenesis and lymphangiogenesis. Front Physiol.

[18] Welford AF, Biziato D, Coffelt SB, Nucera S, Fisher M, Pucci F, Di Serio C, Naldini L, De Palma M, Tozer GM, Lewis CE (2011). TIE2-expressing macrophages limit the therapeutic efficacy of the vascular-disrupting agent combretastatin A4 phosphate in mice. J Clin Invest.

[19] Mazzieri R, Pucci F, Moi D, Zonari E, Ranghetti A, Berti A, Politi LS, Gentner B, Brown JL, Naldini L, De Palma M (2011). Targeting the ANG2/TIE2 axis inhibits tumor growth and metastasis by impairing angiogenesis and disabling rebounds of proangiogenic myeloid cells. Cancer Cell.

[20] Coffelt SB, Tal AO, Scholz A, De Palma M, Patel S, Urbich C, Biswas SK, Murdoch C, Plate KH, Reiss Y, Lewis CE (2010). Angiopoietin-2 regulates gene expression in TIE2-expressing Monocytes and augments their inherent Proangiogenic functions. Cancer Res.

[21] Coffelt SB, Chen YY, Muthana M, Welford AF, Tal AO, Scholz A, Plate KH, Reiss Y, Murdoch C, De Palma M, Lewis CE (2011). Angiopoietin 2 stimulates TIE2-expressing monocytes to suppress T cell activation and to promote regulatory T cell expansion. J Immunol (Baltimore, Md : 1950).

[22] Lewis CE, De Palma M, Naldini L (2007). Tie2-expressing Monocytes and tumor angiogenesis: regulation by hypoxia and Angiopoietin-2. Cancer Res.

[23] De Palma M, Murdoch C, Venneri MA, Naldini L, Lewis CE (2007). Tie2-expressing monocytes: regulation of tumor angiogenesis and therapeutic implications. Trends Immunol.

[24] Hamidullah, Changkija B, Konwar R (2012). Role of interleukin-10 in breast cancer. Breast Cancer Res Treat.

[25] Esquivel-Velazquez M, Ostoa-Saloma P, Palacios-Arreola MI, Nava-Castro KE, Castro JI, Morales-Montor J (2015). The role of cytokines in breast cancer development and progression. J Interf Cytokine Res.

[26] Mocellin S, Marincola FM, Young HA (2005). Interleukin-10 and the immune response against cancer: a counterpoint. J Leukoc Biol.

[27] Forget MA, Voorhees JL, Cole SL, Dakhlallah D, Patterson IL, Gross AC, Moldovan L, Mo X, Evans R, Marsh CB, Eubank TD (2014). Macrophage Colony-stimulating factor augments Tie2-expressing Monocyte differentiation, Angiogenic function, and recruitment in a mouse model of breast cancer. PLoS One.

[28] Owen MR, Sherratt JA (1998). Modelling the macrophage invasion of turnours: effects on growth and composition. IMA J Math Appl Med.

[29] Owen MR, Sherratt JA (1999). Mathematical modelling of macrophage dynamics in tumours. Math Mod Meth Appl S.

[30] Byrne HM, Cox SM, Kelly CE (2004). Macrophage-tumour interactions: in vivo dynamics. Discrete Continuous Dynamical Syst - Series B.

[31] Owen MR, Byrne HM, Lewis CE (2004). Mathematical modelling of the use of macrophages as vehicles for drug delivery to hypoxic tumour sites. J Theor Biol.

[32] Webb SD, Owen MR, Byrne HM, Murdoch C, Lewis CE (2007). Macrophage-based anti-cancer therapy: Modelling different modes of tumour targeting. Bull Math Biol.

[33] Owen MR, Stamper IJ, Muthana M, Richardson GW, Dobson J, Lewis CE, Byrne HM (2011). Mathematical modeling predicts synergistic antitumor effects of combining a macrophage-based, hypoxia-targeted gene therapy with chemotherapy. Cancer Res.

[34] Chen D, Bobko AA, Gross AC, Evans R, Marsh CB, Khramtsov VV, Eubank TD, Friedman A. Involvement of tumor macrophage HIFs in chemotherapy effectiveness: mathematical modeling of oxygen, pH, and glutathione. PLoS One. 2014;9(10) doi: 10.1371/journal.pone.0107511. 10.1371/journal.pone.0107511 PMC4189793 25295611

[35] Bocuk D, Krause P, Niebert S, Pukrop T, Beissbarth T, Ghadimi M, Koenig S (2015). Mouse models of colorectal and mammary cancer liver metastases and microenvironmental interplay with tumor-associated macrophages (TAMs). Z Gastroenterol.

[36] Leonard F, Curtis LT, Ware MJ, Nosrat T, Liu X, Yokoi K, Frieboes HB, Godin B (2017). Macrophage polarization contributes to the anti-Tumoral efficacy of Mesoporous Nanovectors loaded with albumin-bound Paclitaxel. Front Immunol.

[37] Macklin P, McDougall S, Anderson ARA, Chaplain MAJ, Cristini V, Lowengrub J (2009). Multiscale modelling and nonlinear simulation of vascular tumour growth. J Math Biol.

[38] van de Ven AL, Wu M, Lowengrub J, McDougall SR, Chaplain MA, Cristini V, Ferrari M, Frieboes HB (2012). Integrated intravital microscopy and mathematical modeling to optimize nanotherapeutics delivery to tumors. AIP Adv.

[39] Wu M, Frieboes HB, McDougall SR, Chaplain MAJ, Cristini V, Lowengrub J (2013). The effect of interstitial pressure on tumor growth: coupling with the blood and lymphatic vascular systems. J Theor Biol.

[40] Lewis C, Murdoch C (2005). Macrophage responses to hypoxia : implications for tumor progression and anti-cancer therapies. Am J Pathol.

[41] McDougall SR, Anderson ARA, Chaplain MAJ (2006). Mathematical modelling of dynamic adaptive tumour-induced angiogenesis: clinical implications and therapeutic targeting strategies. J Theor Biol.

[42] Pries AR, Secomb TW, Gaehtgens P (1998). Structural adaptation and stability of microvascular networks: theory and simulations. Am J Phys.

[43] McDougall SR, Anderson AR, Chaplain MA, Sherratt JA (2002). Mathematical modelling of flow through vascular networks: implications for tumour-induced angiogenesis and chemotherapy strategies. Bull Math Biol.

[44] Pries AR, Hopfner M, le Noble F, Dewhirst MW, Secomb TW (2010). The shunt problem: control of functional shunting in normal and tumour vasculature. Nat Rev Cancer.

[45] van de Ven AL, Wu M, Lowengrub J, McDougall SR, Chaplain MAJ, Cristini V, Ferrari M, Frieboes HB. Integrated intravital microscopy and mathematical modeling to optimize nanotherapeutics delivery to tumors. AIP Adv. 2012;2(1) doi: 10.1063/1.3699060. 10.1063/1.3699060 PMC3321519 22489278

[46] Spinney L (2006). Caught in time. Nature.

[47] Cui Y-LL, Hui-Kai, Zhou H-Y, Zhang T, Li Q (2013). Correlations of tumor-associated macrophage subtypes with liver metastases of colorectal cancer. Asian Pac J Cancer Prevent.

[48] Frieboes HB, Curtis LT, Wu M, Kani K, Mallick P (2015). Simulation of the protein-shedding kinetics of a fully vascularized tumor. Cancer Inform.

[49] Kozlowski L, Zakrzewska I, Tokajuk P, Wojtukiewicz MZ (2003). Concentration of interleukin-6 (IL-6), interleukin-8 (IL-8) and interleukin-10 (IL-10) in blood serum of breast cancer patients. Rocz Akad Med Bialymst.

[50] Macklin P, Lowengrub J (2007). Nonlinear simulation of the effect of microenvironment on tumor growth. J Theor Biol.

[51] Macklin P, Lowengrub JS (2008). A new ghost cell/level set method for moving boundary problems: application to tumor growth. J Sci Comput.

[52] Frieboes HB, Jin F, Chuang YL, Wise SM, Lowengrub JS, Cristini V (2010). Three-dimensional multispecies nonlinear tumor growth-II: tumor invasion and angiogenesis. J Theor Biol.

[53] Quatromoni JG, Eruslanov E (2012). Tumor-associated macrophages: function, phenotype, and link to prognosis in human lung cancer. Am J Transll Res.

[54] Williams CB, Yeh ES, Soloff AC (2016). Tumor-associated macrophages: unwitting accomplices in breast cancer malignancy. NPJ Breast Cancer.

[55] Vasudev NS, Reynolds AR (2014). Anti-angiogenic therapy for cancer: current progress, unresolved questions and future directions. Angiogenesis.

[56] de Groot JF, Fuller G, Kumar AJ, Piao Y, Eterovic K, Ji Y, Conrad CA (2010). Tumor invasion after treatment of glioblastoma with bevacizumab: radiographic and pathologic correlation in humans and mice. Neuro-Oncology.

[57] Lamszus K, Kunkel P, Westphal M (2003). Invasion as limitation to anti-angiogenic glioma therapy. Acta Neurochir Suppl.

[58] Paez-Ribes M, Allen E, Hudock J, Takeda T, Okuyama H, Vinals F, Inoue M, Bergers G, Hanahan D, Casanovas O (2009). Antiangiogenic therapy elicits malignant progression of tumors to increased local invasion and distant metastasis. Cancer Cell.

[59] Pennacchietti S, Michieli P, Galluzzo M, Mazzone M, Giordano S, Comoglio PM (2003). Hypoxia promotes invasive growth by transcriptional activation of the met protooncogene. Cancer Cell.

[60] Cristini V, Frieboes HB, Gatenby R, Caserta S, Ferrari M, Sinek J (2005). Morphologic instability and cancer invasion. Clin Cancer Res.

[61] Cristini V, Lowengrub J, Nie Q (2003). Nonlinear simulation of tumor growth. J Math Biol.

[62] Frieboes HB, Zheng X, Sun CH, Tromberg B, Gatenby R, Cristini V (2006). An integrated computational/experimental model of tumor invasion. Cancer Res.

[63] Mantovani A, Allavena P (2015). The interaction of anticancer therapies with tumor-associated macrophages. J Exp Med.

[64] Curtis LT, England CG, Wu M, Lowengrub J, Frieboes HB (2016). An interdisciplinary computational/experimental approach to evaluate drug-loaded gold nanoparticle tumor cytotoxicity. Nanomedicine (Lond).

[65] Curtis LT, Wu M, Lowengrub J, Decuzzi P, Frieboes HB (2015). Computational modeling of tumor response to drug release from vasculature-bound Nanoparticles. PLoS One.

[66] Wu M, Frieboes HB, Chaplain MA, SR MD, Cristini V, Lowengrub JS (2014). The effect of interstitial pressure on therapeutic agent transport: coupling with the tumor blood and lymphatic vascular systems. J Theor Biol.

[67] Gordon SR, Maute RL, Dulken BW, Hutter G, George BM, McCracken MN, Gupta R, Tsai JM, Sinha R, Corey D, Ring AM, Connolly AJ, Weissman IL (2017). PD-1 expression by tumour-associated macrophages inhibits phagocytosis and tumour immunity. Nature.

[68] Nugent LJ, Jain RK (1984). Extravascular diffusion in normal and neoplastic tissues. Cancer Res.

